# Seasonal prevalence of bacteria in the outflow of two full-scale municipal wastewater treatment plants

**DOI:** 10.1038/s41598-023-37744-3

**Published:** 2023-06-30

**Authors:** Magdalena Domańska, Magdalena Kuśnierz, Katarzyna Mackiewicz

**Affiliations:** Institute of Environmental Engineering, Wroclaw University of Environmental and Life Sciences, pl. Grunwaldzki 24, 50-363 Wrocław, Poland

**Keywords:** Environmental impact, Microbial ecology

## Abstract

Despite many modern wastewater treatment solutions, the most common is still the use of activated sludge (AS). Studies indicate that the microbial composition of AS is most often influenced by the raw sewage composition (especially influent ammonia), biological oxygen demand, the level of dissolved oxygen, technological solutions, as well as the temperature of wastewater related to seasonality. The available literature mainly refers to the relationship between AS parameters or the technology used and the composition of microorganisms in AS. However, there is a lack of data on the groups of microorganisms leaching into water bodies whose presence is a signal for possible changes in treatment technology. Moreover, sludge flocs in the outflow contain less extracellular substance (EPS) which interferes microbial identification. The novelty of this article concerns the identification and quantification of microorganisms in the AS and in the outflow by fluorescence in situ hybridization (FISH) method from two full-scale wastewater treatment plants (WWTPs) in terms of 4 key groups of microorganisms involved in the wastewater treatment process in the context of their potential technological usefulness. The results of the study showed that Nitrospirae, Chloroflexi and *Ca*. Accumulibacter phosphatis in treated wastewater reflect the trend in abundance of these bacteria in activated sludge. Increased abundance of betaproteobacterial ammonia-oxidizing bacteria and Nitrospirae in the outflow were observed in winter. Principal component analysis (PCA) showed that loadings obtained from abundance of bacteria in the outflow made larger contributions to the variance in the PC1 factorial axis, than loadings obtained from abundance of bacteria from activated sludge. PCA confirmed the reasonableness of conducting studies not only in the activated sludge, but also in the outflow to find correlations between technological problems and qualitative and quantitative changes in the outflow microorganisms.

## Introduction

The role of wastewater treatment plants (WWTPs) is to protect rivers and water bodies from the influx of pollutants resulting from industrial activities and those associated with human hygiene and sanitation^1^. For this purpose, new technologies for wastewater treatment are being implemented, such as nanotechnologies, including filtration processes, mainly nanofiltration^2^, adsorption processes using different nanomaterials or nano-absorbents, and photocatalytic degradation processes with or without photocatalysts^3^, as well as the use of natural materials^4^. Despite many modern solutions, the activated sludge (AS) process is still the one most often used. The bacteria associated with the flocs conduct nitrification and denitrification processes to oxidize the nitrogen compounds, high concentrations of which adversely affect the microflora of the receiver. A complete understanding of nutrient removal requires the identification of key microorganisms responsible for the transformation of substrates in the various phases of treatment, although efforts are being made to distinguish characteristic bacteria whose presence is a signal for possible changes in treatment technology. The prevalence of molecular methods such as fluorescence in situ hybridization (FISH) and polymerase chain reaction (PCR) has helped researchers understand that the structure of activated sludge is more complex^5^. The microbial diversity uncovered by molecular methods (including next-generation sequencing (NGS)) is enormous. According to Zhang et al.^6^ there are more than 700 genera and thousands of operational taxonomic units (OTUs). Research by Zhang et al.^1^ indicated that the bacterial community is determined by the influent wastewater content, the degree of sludge aeration, the technological solution, the location of the treatment plant and seasonality. Also, a study by Xu et al.^7^ showed that factors such as biological oxygen demand (BOD5), dissolved oxygen (DO), water temperature, and influent ammonia had the greatest impact on the microflora composition, while a study by Kang et al.^8^ indicated that seasonal changes affected DO and temperature significantly. In contrast, a study by Nielsen et al.^9^ conducted on 25 full-scale WWTPs showed that despite the use of different technologies and composition of raw wastewater, the sludge was characterized by a limited composition of key species, and the variable was primarily the number of microorganisms. This is consistent with a study of nitrifying bacteria diversity carried out by Siripong and Rittman^10^ on seven wastewater treatment plants, which showed that despite differences in the influent flow rate, plant size, solids retention time (SRT) or wastewater temperature, ammonia oxidizing bacteria (AOB) such as *Nitrosomonas europaea/eutropha, Nitrosomonas oligotropha, Nitrosomonas communis* and *Nitrosospira*, as well as nitrite oxidizing bacteria (NOB) such as *Nitrobacter* and *Nitrospira* were observed at all WWTPs. The study found that only the seasonal variation in temperature affected the diversity of nitrifying bacteria, especially between *Nitrosospira* and *Nitrosomonas*. The extensive meta-analysis conducted on data from 50 WWTPs in 13 countries has confirmed that the factor with the most influence on the microbial structure was climate type^11^. It should be noted, however, that a change in technological conditions can increase bacterial activity, which does not necessarily manifest itself as an increase in the number of bacteria^12^. Research by Petrovski et al.^13^ showed that temperature and influent composition exert significant influence on the variation of bacteria in AS, while Shchegolkova et al.^14^ found that the chemical rather than bacterial composition of incoming wastewater was the main factor in the formation of microbial structure in the activated sludge. Research by Valentin-Vargas et al.^15^ shows that large wastewater treatment plants have a more diverse bacterial community, while changes in structure are not as dynamic as in smaller reactors. On the other hand, Isazadeh et al.^16^ demonstrated that the size of the treatment plant does not matter for population stability. Despite the interest in the topic, so far there have been a few technical-scale studies that take into account multiannual data from which seasonal variation can be observed^1,17–25^.The publications mainly refer to the relationship between wastewater parameters or the technology used on the one hand, and the composition of microorganisms in the activated sludge on the other. However, there is a lack of data on the groups of microorganisms that are leaching into water bodies and their importance in the wastewater treatment process. It is well known that the quality of wastewater treatment depends on the presence of protozoa (ciliates, flagellates, amoebae) in activated sludge^26^. Ciliates are widely known for feeding on free floating bacteria, initiating floc formation and grazing effect^27^. The higher number of protozoa, the lower percentage of floc groups less than 10 μm in activated sludge^28^. Crawling ciliates contribute to bioaggregation in activated sludge and stabilizing interactions between cells and surfaces and also between cells^29^. Although the formation of clusters by bacteria protects them from being leached out of the reactor or attacked by protozoa, there is always the risk of nitrifiers leaching out of the system, leading to a breakdown of the nitrification process^12^. It should also be mentioned that the phenomenon of leaching of nitrifiers can be associated with the procedure of adding bacterial inoculum to the aeration chamber for improving nitrification efficiency^30^. Earlier studies suggest that much smaller flocs may contain the bacteria responsible for treatment processes entering the outflow^31^. A study by Shomar et al.^32^ using next-generation sequencing (NGS) has confirmed that at various stages of treatment, a dominance of Proteobacteria is observed. The authors suggest that it would be desirable to identify also living, metabolically active microorganisms, however, the occurrence of particular groups in the outflow with the wastewater treatment process were not taken into account. Currently, research on the outflow, in particular, focus on the phenomenon of the spread of pathogens, microplastics and antibiotic-resistant bacteria^33,34^. The novelty of this article concerns the similarities and differences in the sludge taken from activated sludge and the outflow in terms of the identification of microorganisms from two full-scale WWTPs using the FISH method for 4 key groups of microorganisms (betaproteobacterial ammonia-oxidizing bacteria, Nitrospirae, Chloroflexi, and *Candidatus* Accumulibacter phosphatis) involved in the wastewater treatment process in the context of their potential technological usefulness.

### Description of the wastewater treatment plants

**Figure 1:**
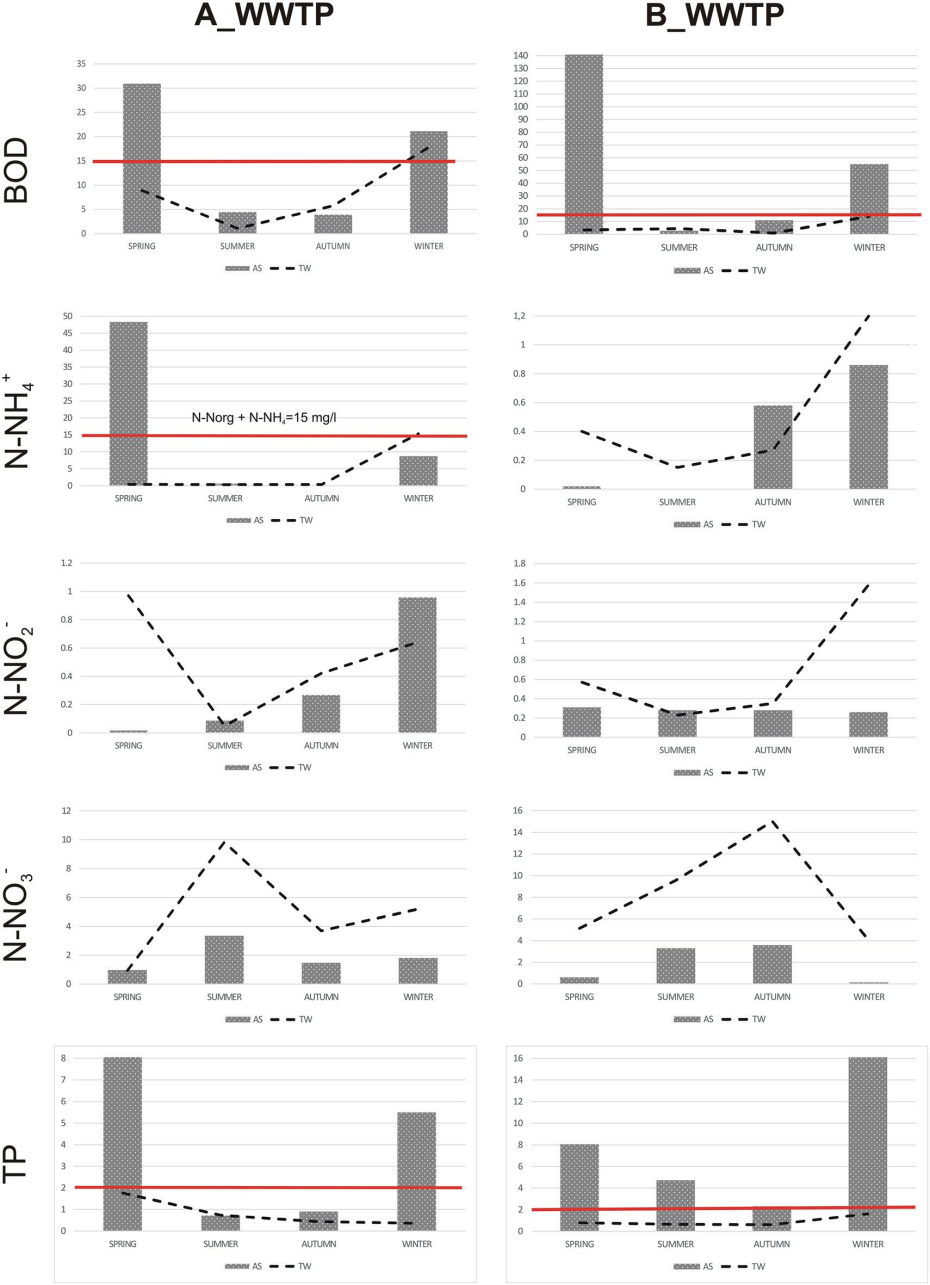
Seasonal changes in BOD, N-NH_4_^+^, NNO_2_^−^, NNO_3_^−^ and TP in activated sludge (AS) and treated wastewater (TW) in A and B-WWTP. Compound concentrations are presented in mg per liter. Red line indicates the permissible level in Poland.

Two mechanical-biological wastewater treatment plants with activated sludge were selected for the study. The first treatment plant, labeled A-WWTP, showed some problems related to the implementation of integrated fixed film activated sludge (IFAS) technology. The other one, marked as B-WWTP, had no significant problems in terms of basic treatment parameters (nitrogen, phosphorus, suspended solids, BOD and COD). Both WWTPs are located in Poland, near the capital of the Lower Silesia province. A and B-WWTPS are located respectively 12 and 75 km from Wroclaw. A-WWTP started operating in 2009, while B-WWTP has undergone a number of modernizations, the most recent of which was carried out in the spring of 2021 and involved the use of IFAS technology. Biological treatment of wastewater at the A-WWTP is carried out in nitrification, denitrification and defosfatation chambers according to the Anaerobic-anoxic-aerobic (A2O) method, and the wastewater treatment process is assisted by dosing polyaluminum chloride to remove phosphorus. Biological treatment of wastewater at the B-WWTP is carried out using the activated sludge method according to the modified Bardenpho system, which involves secondary denitrification of the sludge and allows wastewater to be treated for phosphorus compounds without the use of additional chemicals. The average capacity of the A-WWTP is 2300 m^3^/d, while an average of about 600 m^3^ currently flows through the B-WWTP on a daily basis. Sewage flows into each WWTP through the sewer system and is delivered by vacuum trucks. The receiver of treated wastewater for both A and B-WWTP is a river. The pH at both WWTPs was in a similar range of 7.6–8.1 in the activated sludge from A-WWTP and 7.7–8.16 in the activated sludge from B-WWTP. At the outflow, the pH was slightly higher, ranging from 7.4 to 9.2 in the treated outflow from A-WWTP and from 8.6 to 8.75 for B-WWTP. The temperature of the activated sludge from the A-WWTP along with the average monthly temperatures for Wroclaw are shown in Fig. S1 (see Supplementary material). The activated sludge temperature was not tested at B-WWTP. It is not mandatory at small wastewater treatment plants. The average monthly temperatures from 2000 to 2022 did not vary significantly. Wastewater temperatures are typically 5–10 °C higher than the air temperature in winter, with both having similar temperatures in summer. The parameters of wastewater that flows into the treatment plants are shown in Table 1. Figure 1 depicts seasonal changes in BOD, N-NH_4_^+^, NNO_2_^−^, NNO_3_^−^ and TP in activated sludge and treated wastewater in A and B-WWTP. Orthophosphate concentration varied from 0.05 to 0.45 mg/L for A-WWTP and from 0.02 to 2.26 mg/L for B-WWTP in AS, while at the outflow in the range of 0–0.26 mg/L and 0.13–0.88 mg/L for treatment plants A and B, respectively.

**Table 1:** The WWTPs parameters and the average load of inflowing pollutants.

WWTP	The type of influent	Process	Flow	pH	BOD	COD	TN	N-NH_4_	TP	Sulfate	Chlorides
			(m^3^/d)		(mgO_2_/L)			(mg/L)			
A	Domestic sewage predominant	A2O + fixed bed	2300	7.83	397.3	1057.0	104.3	72.70	10.57	101.08	352.99
B	Domestic sewage predominant	Modified Bardenpho	600	7.52	53.1	98.03	33.57	18.71	20.70	192.53	140.10

## Methods

Two liter samples of activated sludge (AS) and treated wastewater (TW) for A and B WWTP were collected on every season and subjected to physicochemical and FISH analyses as well as SVI calculation. The tests were done in the laboratory of Wrocław University of Environmental and Life Sciences. The following standards were used for the various parameters tested: BOD5—PN-EN 1899-1:2002; COD—PN-ISO 6060:2006; Total suspension—PN-EN 872:2002; N-NH4^+^—PN-ISO 7150-1:2002; N-NO_2_^−^—PN-EN 26777:1999; N-NO_3_^−^—PN-82/C-04576.08; Total nitrogen (TN) and Organic nitrogen (Norg)—calculation method, Total phosphorus and orthophosphates (ortho-P)—PN-EN ISO 6878:2006; Turbidity (TUR)—PN-EN ISO 7027:2003. The sludge volume index (SVI) test was conducted after settling the total suspension of the mixed liquid for 30 min in a 1 L cylinder.

### FISH and quantitative analysis

**Figure 2:**
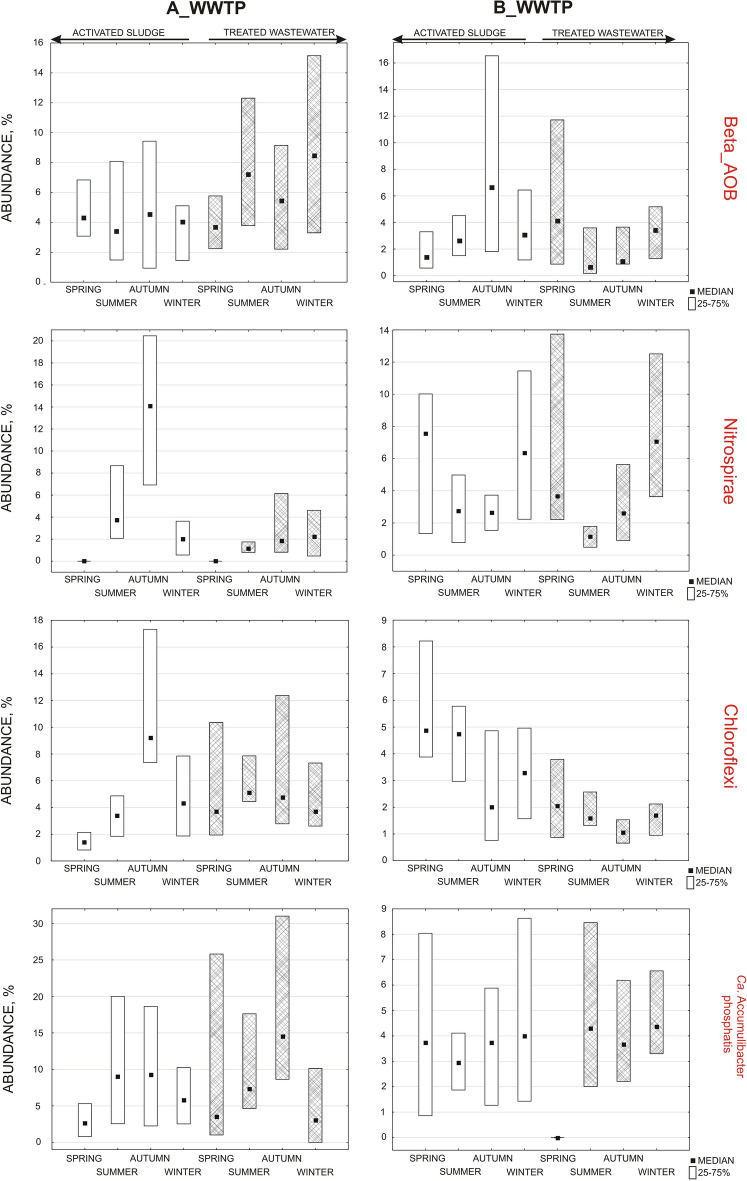
Seasonal changes in the abundance of betaproteobacterial ammonia-oxidizing bacteria, Nitrospirae, Chloroflexi and *Ca.* Accumulibacter phosphatis in activated sludge and treated wastewater in A and B-WWTP.

Four groups of microorganisms, all of which crucial to aerobic activated sludge treatment plants, were selected for the study, namely betaproteobacterial ammonia-oxidizing bacteria, Nitrospirae, *Candidatus* Accumulibacter phosphatis, and Chloroflexi. To identify them, the FISH method and oligonucleotide probes listed in Table S1 (see Supplementary material) were used. At first activated sludge (AS)and treated wastewater (TW) were fixed with 4% paraformaldehyde (PFA) and then the standard protocol according to Amann was used in the research^12^. For the quantitative analysis, 25 images, each of the sediment from the outflow stained with general probe, specific probe and DAPI reagent, were taken sequentially. A total of 800 images were taken for the activated sludge and 800 for the sediment from the treated wastewater (see Supplementary Figs. S2a,b, S3a,b). A Nikon Eclipse Ni-E C2 confocal microscope equipped with UV-2A, B-2A and G-2A filters (with wavelengths of excitation (EX) 330–380, 450–490 and 510–560 nm, respectively) and Nis-Elemnets AR 4.30 software were used for the analysis. The appropriate threshold (the level of fluorescence corresponding to the presence of the probe's signal) was then defined for each probe. In this procedure, binary images (see Supplementary Figs. S2c,d, S3c,d) were obtained for which fluorescent areas were automatically counted. After determining the area of fluorescence, the percentage ratio of the specific probe area to DAPI (VECTASHIELD^®^ Mounting Medium with DAPI reagent, Vector lab, catalog number: H-1200-10) was calculated^31,35^. In this way, 32 different percentage ratios were obtained for AS (4 microorganisms × 4 seasons × 2 WWTPs) and 32 for TW, for which medians and percentiles of 25–75% were defined in Statistica 13.3. Minimal and maximum values were excluded from the Fig. 2. The sludge was diluted at a ratio of 1:10 and 1:30 for the TW and AS respectively. The results in the Fig. 2 do not contain the information about the dilution.

### PCA analysis

Principal Component Analysis (PCA) is a commonly used method to determine interactions between variables^36^. The number of initial variables was reduced using Kaiser's criterion, which requires that the factors being analysed have eigenvalues higher than 1. Principal component analysis is regarded as suitable for variables displaying some linearity, which is why it is believed that if the average absolute value of the correlation coefficient is lower than 0.3, the results of factor analysis fail to adequately describe the relations between particular variables. Firstly, the results of the quantitative analysis in the form of medians (Beta AOB _AS, Beta AOB_TW, Ntspa_AS, Ntspa_TW, Chloroflexi_AS, Chloroflexi_TW, PAO_AS, PAO_TW) for both activated sludge (AS) and treated wastewater (TW) by season were considered for PCA. In addition to the data from the quantitative analysis, results from the analysis of all physical and chemical parameters (BOD, COD, TN, Norg, N-NH_4_^+^, N-NO_3_, N-NO_2_^−^, pH, TP, ortho-P, Turbidity, COD/BOD, COD/N, SVI) were also included. To find the relationship between the independent variables, the nonlinear estimation module incorporated in STATISTICA 13.3 was made. Usually the number of resulting eigenvectors (Principal Components) is equal to the number of variables. Since, the cumulative eignevalue [%] for factor 7 reached 100.0000%, factor 8 was not included in the Tables 2 and 5.

**Table 2:** Eigenvalues of the correlation matrix for quantitative analysis results by season.

Factor	Eigenvalue	Cumulative eigenvalue%	Cumulative eigenvalue	Cumulative %
**1**	**3.077882**	**38.47353**	**3.077882**	**38.4735**
**2**	**1.989289**	**24.86611**	**5.067171**	**63.3396**
**3**	**1.378957**	**17.23696**	**6.446128**	**80.5766**
4	0.894711	11.18389	7.340840	91.7605
5	0.406721	5.08402	7.747561	96.8445
6	0.208520	2.60650	7.956081	99.4510
7	0.043919	0.54899	8.000000	100.0000

Factors for which cumulative eigenvalues exceeded 80 % [bold].

### Ethics approval

This research did not involve human participants and/or animals.

## Results and discussion

### Ammonia oxidizing bacteria (AOB)

The results of the study of betaproteobacterial ammonia-oxidizing bacteria (beta AOB) showed that for A-WWTP, higher abundances were observed in winter and summer, while for B-WWTP, most of these bacteria in the outflow were observed in spring and winter, and no significant leaching of nitrifiers was observed in other seasons (Fig. 2). The increase in beta AOB at the outflow in the summer for A-WWTP was probably due to the implemented new technology, where technological problems were observed during this period^37^. The composition of activated sludge fed with municipal wastewater is usually dominated by Proteobacteria, with Betaprotebacteria being the dominant class^15^. In the study of Gao et al.^17^, Betaproteobacteria accounted for 42.9–63.0% in the sediment, and in the study of Xu et al.^7^ from 34.4 to 65.8%. According to Xia et al.^38^, nitrifiers accounted for 5.3–11.5% of the total bacteria. Although studies conducted in regions with lower temperatures (Finland, Polar Arctic Circle Region) indicate that the bacterial composition varies significantly^19^. Griffin and Wells^20^ found that *Nitrosomonas* showed little seasonal variation. Studies by Ju et al.^22^ confirmed a significantly higher abundance of *Nitrosomonas* in the sludge during summer. Research by Ju and Zhang^23^ indicates that *Nitrosomonas* has high transcriptional activity, and despite its low abundance in the activated sludge it can carry out ammonia oxidation. The authors suggest that during the decrease in AOB activity, the process may be carried out by AOB yet to be identified. Therefore, it can be noticed that the result of changes in bacteria abundance at the outflow may suggest nitrification problems, which occurred in the activated sludge chamber. The increase in BOD and N-NH4^+^ concentrations in the outflow reflected the increase in beta AOB in the outflow at this time (Fig. 1). This confirms the research by Johnston et al.^21^ that seasonal breakdowns in nitrification occur mainly during the winter months.

### Nitrospirae

An increase in the abundance of Nitrospirae in the outflow was observed in winter for both treatment plants (Fig. 2). For A-WWTP, greater abundance in AS was observed in summer and autumn, and for B in spring and winter, reflecting changes in the TW. In the study by Ju et al.^22^, Nitrospirae showed a significantly higher abundance in activated sludge in summer, whereas in the treatment plants A and B, Nitrospirae’s abundance was lowest at the outflow in summer. According to a study by Griffin and Wells^20^, Nitrospira dominated during the summer months in all reactors, and its growth was related to temperature and the nitrite content of the activated sludge. Probably for this reason, at the beginning of the process, Nitrospirae was not observed in the spring in activated sludge samples and in treated wastewater of A-WWTP due to very low nitrate(III) levels (Fig. 1). The study showed that the dominant microorganism responsible for nitrate(III) oxidation in most wastewater treatment plants is not *Nitrobacter* spp. but uncultured *Nitrospira-like*^39^. *Nitrospira* has the ability to perform complete nitrification, called comammox (COMplete AMMonia OXidation)^40^. In the study by Xu et al.^7^, *Nitrospira* was present in all wastewater samples, while research by Zhang et al.^41^ indicated that *Nitrospira* dominated in municipal wastewater. Pilot-scale studies in a sequencing batch biofilm reactor showed that *Nitrospira* bacteria were observed in clusters together with AOB bacteria and heterotrophic bacteria^40^. AOB (*Nitrosomonas*) also co-occurs with NOB (*Nitrospira*) in a mutualistic symbiosis relationship, in which AOB provides nitrite for NOB, and in return NOB removes nitrite to prevent its inhibition on AOB^23^. Ju and Zhang^23^ showed in a five-year analysis that the Nitrospira class tended to be more persistent than others. Comammox nitrifiers have a selective advantage over other nitrifiers under stressful conditions, especially at low DO levels^42^. *Nitrospira* is a slower-growing K-strategist, meaning that it prefers a low-nitrite environment^12^, although some Nitrospira phylotypes can vary in nitrite tolerance^43^. *Nitrospira* is not always the core organism of NOB. A study by Saunders et al.^25^ found that the genus *Nitrotoga* had a higher read abundance than *Nitrospira* in nine samples. Based on the results of the wastewater inflow, the researchers suggest that the selection process may be taking place as early as in the sewers, or it is only at the wastewater treatment plant that selection takes place, depending on the technological conditions. Studies have indicated that their selection depends on temperature, and *Nitrotoga* tends to occur at lower temperatures than *Nitrospira*^44^. This may explain the fact that at lower temperatures other species dominate and Nitrospirae goes excessively into the outflow. However, this needs to be further investigated. Nevertheless, according to a study by Zhang et al.^1^, *Nitrospira*, as the third dominant microorganism in the aerobic sludge, appeared only in winter. *Nitrospira* was slightly more abundant in those systems that had higher concentrations of influent ammonia^17^. Considering the concentration of ammonia nitrogen in the raw wastewater, much higher concentrations were observed for treatment plant A at an average of 72.7 mg/L N-NH_4_^+^, while for treatment plant B they did not exceed 30 mg/L N-NH_4_^+^ during the studied periods. For A-WWTP, ammonium nitrogen on the influent did not vary significantly during the periods analyzed, and Nitrospirae abundance was observed most in the AS in autumn. Ammonium nitrogen concentration in the inflow for B-WWTP was the highest in spring, which coincided with the highest abundance of Nitrospirae in the AS.

### Chloroflexi

The results of the experiments showed that Chloroflexi dominated in the outflow in summer especially for A-WWTP, while in B-WWTP they were most abundant in spring, although the results for B-WWTP were not as varied as for A-WWTP (Fig. 2). Because Chloroflexi are photophilic^45^ they will probably tend to accumulate on the outside of the floc or in the water tone, which exposes them to greater mechanical damage, such as that associated with aeration of activated sludge, and will cause them to be leached more from the sludge tanks. The operation of A-WWTP was more variable compared to B-WWTP due to the implementation of new technology. Filamentous bacteria, which include Chloroflexi, not only give cohesiveness to the floc structure (stabilize sediment flocs)^46^, but also hydrolyze proteins, lipids and polysaccharides, and oxidize carbon, sulfur and iron compounds. Chloroflexi are capable of degrading polymers and complex organics, take polysaccharides and proteins from EPS and dead cells, and decompose them into simple organic compounds that can be metabolized by other bacterial species^41,46^. Excessive filamentous growth is not beneficial to the operation of the treatment plants, as it causes worse sludge settling, which in turn increases the amount of sludge leaching into the outflow and decreases the quality of treated wastewater. It also negatively affects the process of sludge recirculation and problems with excess sludge management. Chloroflexi are metabolically versatile and responsible for the breakdown of carbohydrates under aerobic and anaerobic conditions^47^. Recent studies suggest that Chloroflexi bacteria may be involved in the denitrification process^48^. Identification of filamentous microorganisms is not easy, as classification into one morphotype is not synonymous with similar physiology. Often bacteria can be sensitive to other factors that inhibit their growth. According to the study, Chloroflexi in activated sludge were present in an abundance of 2–22, 3–35%^49^, whereas in the study by Zhang et al.^6^ Chloroflexi accounted for 8.7%. The results of the principal component analysis (PCA) showed that an increase in SRT and N-NO_3_^−^ concentrations could promote the accumulation of the OTU associated with Chloroflexi^23^. The studies of Zhang et al.^1^ and Kang et al.^8^ confirmed that members of Chloroflexi dominated in aerobic activated sludge during summer. Similar results were noted for at the B-WWTP. What is the most surprising, is the tendency between AS and TW in both WWTPs.

**Figure 3:**
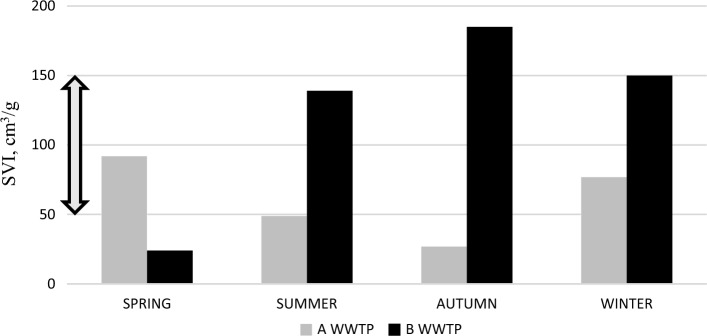
Seasonal changes in the sludge volume index (SVI) at treatment plant (**A**) and (**B**). The grey arrow indicates the optimal range for SVI.

High values of the sludge volume index (SVI) indicate sludge swelling and are usually associated with an increased abundance in filamentous bacteria. At B-WWTP, higher SVI values were observed in summer, autumn and winter, and a low abundance of Chloroflexi was observed in the outflow during this time, while the highest number of these bacteria was identified in spring when the index was low. A similar situation occurred at A-WWTP where low SVI indices in summer and autumn corresponded with a higher abundance of Chloroflexi bacteria in the outflow (Fig. 3). Sludge bulking occurs when aggregates form a non-compact and low density flocs, when large contents of filamentous bacteria occur or when an excessive amount of extracellular polysaccharides (EPS) is provided. In case of WWTP-B high SVI values (> 150 mL/g) while low proliferation of Chloroflexi may indicate the occurrence of viscous bulking instaed of filamentous bulking. The mode of bulking changed between the viscous bulking and filamentous bulking depending on such factors as nutrient deficiency of wastewater inflow^50^ and the presence of different cations^51^. In this case (WWTP-B) Chloroflexi was not responsible for the high SVI values.

### Polyphosphate-accumulating organisms (PAO)

According to the literature PAO bacteria form clusters, which protects them from leaching out of the reactor^49^. Research results indicate that these bacteria are also leached from biological reactors. At A-WWTP, the highest abundance of bacteria in the outflow was observed in autumn, the same as in the activated sludge (Fig. 2). In the outflow of B-WWTP, bacteria were scarce compared to the A-WWTP and there was no clear variation between samples. The similar was observed in activated sludge (Fig. 2). Successful operation of the phosphorus removal process by this bacteria requires separation of systems into anaerobic and aerobic, although aerobic conditions can also run the process but must be maintained at low dissolved oxygen (DO). Such a system is found at the A-WWTP, where aerobic conditions of about 2 mg/L are maintained in the activated sludge chamber, while low-oxygen or anaerobic conditions are maintained in the fibers (fixed-bed). In B-WWTP, the system is not supported by additional phosphorus precipitation, but secondary denitrification takes place. Removal of phosphorus compounds is supported by phosphate-accumulating organisms (PAO) or glycogen-accumulating organisms (GAO)^52^. PAO under alternating aerobic-anaerobic conditions take up phosphorus in an inorganic form in excess of their metabolic requirements and accumulating it in intracellular polyphosphate (polyP). Under anaerobic conditions, the accumulated poliPis degraded to produce the energy required for the uptake in volatile fatty acids (VFAs), and subsequent release of inorganic phosphorus (Pi) into the rector. The VFAs are then converted to polyhydroxyalkanoate (PHA), which is oxidized under aerobic conditions, and Pi is taken up with the formation of polyP, along with the simultaneous replenishment of glycogen and cell growth^53^. Among the most common PAOs are *Accumulibacter, Tetrasphaera*, and *Halomonas*, which take up volatile fatty acids (VFA), amino acids, and glucose or ethanol, respectively. *Accumulibacter* are more abundant in municipal wastewater compared to industrial wastewater, and their abundance in WWTPs does not exceed 12%^54^. In the review by Izadi et al.^55^, in enhanced biological phosphorus removal (EBPR) systems, the amounts were higher and *Ca.* Accumulibacter identified by FISH occurred from 3 to as much as 85% in acetate-based feed reactors. At both treatment plants, acceptable concentrations of total phosphorus in the outflow were not exceeded (Fig. 1). The highest concentrations in the outflow were observed in spring and winter for treatment plants A and B, respectively. Taking into account the ortho-*P* values, the highest concentrations for treatment plant A were recorded in winter (0.26 mg/L) and for treatment plant B in summer (0.88 mg/L). At the outflow, higher abundance of these bacteria were observed in summer for B-WWTP, while at A-WWTP it was especially in autumn. As with Nitrospirae and Chloroflexi, PAO bacteria in TW also reflect the trend in abundance of these bacteria in AS.

Despite the clear tendency, no statistical significance (*P* > 0.05) was obtained for the differences found between the bacterial abundance data from WWTP A and B, as well as the bacterial abundance data from AS and TW. The data belonging to the same population of data could alternatively be used to draw similar conclusions. In order to analyze the similarities and differences between the AS and TW, a principal component analysis (PCA) was carried out.

### Principal component analysis

Table 2 shows the calculated eigenvalues of the 7 factors determined by the correlation matrix. The factors were ordered as the eigenvalues while the explained variance increased. For this dataset, factor 1 explained 38.47% of the total variance of the dataset, while factor 2 explained 24.86%. The cumulative sum of the explained variance of the first two factors was 63.33%. As for the data from quantitative analyses, there were 3 factors that satisfied the Kaiser criterion, and their cumulative eigenvalues amounted to 80.57%.

Table 3 shows the eigenvectors of the first 3 factors with the highest proportion in the cumulative eigenvalue. The data demonstrate that particular factors are related to different loadings obtained from abundance of bacteria observed in different seasons and WWTPs. In the case of A-WWTP, factor 1 was strongly influenced by loadings obtained from abundance of spring and winter. A very similar result can be observed for B-WWTP where factor 1 depended on loadings obtained from abundance of winter and spring. Factor 2 was under the influence on loadings obtained from abundance of summer and autumn for A-WWTP and summer for B-WWTP. The value of factor 3 strongly correlated with the loadings obtained from abundance of bacteria noted in autumn of B-WWTP.

**Table 3:** Eigenvectors of the first three factors for the set of samples for quantitative analysis results by season (variables).

Variables	Factor 1	Factor 2	Factor 3
Spring_A	**− 0.933495**	− 0.235919	0.105610
Summer_A	**− 0.642875**	0.478167	− 0.451490
Autumn_A	− 0.302055	**0.878955**	− 0.059320
Winter_A	**− 0.779527**	− 0.112924	− 0.577862
Spring_B	**0.717583**	0.422856	− 0.418209
Summer_B	− 0.364956	**0.683396**	0.360865
Autumn_B	− 0.325743	0.210837	**0.721979**
Winter_B	**0.583143**	0.478941	0.011896

Significant values are in [bold].

**Figure 4:**
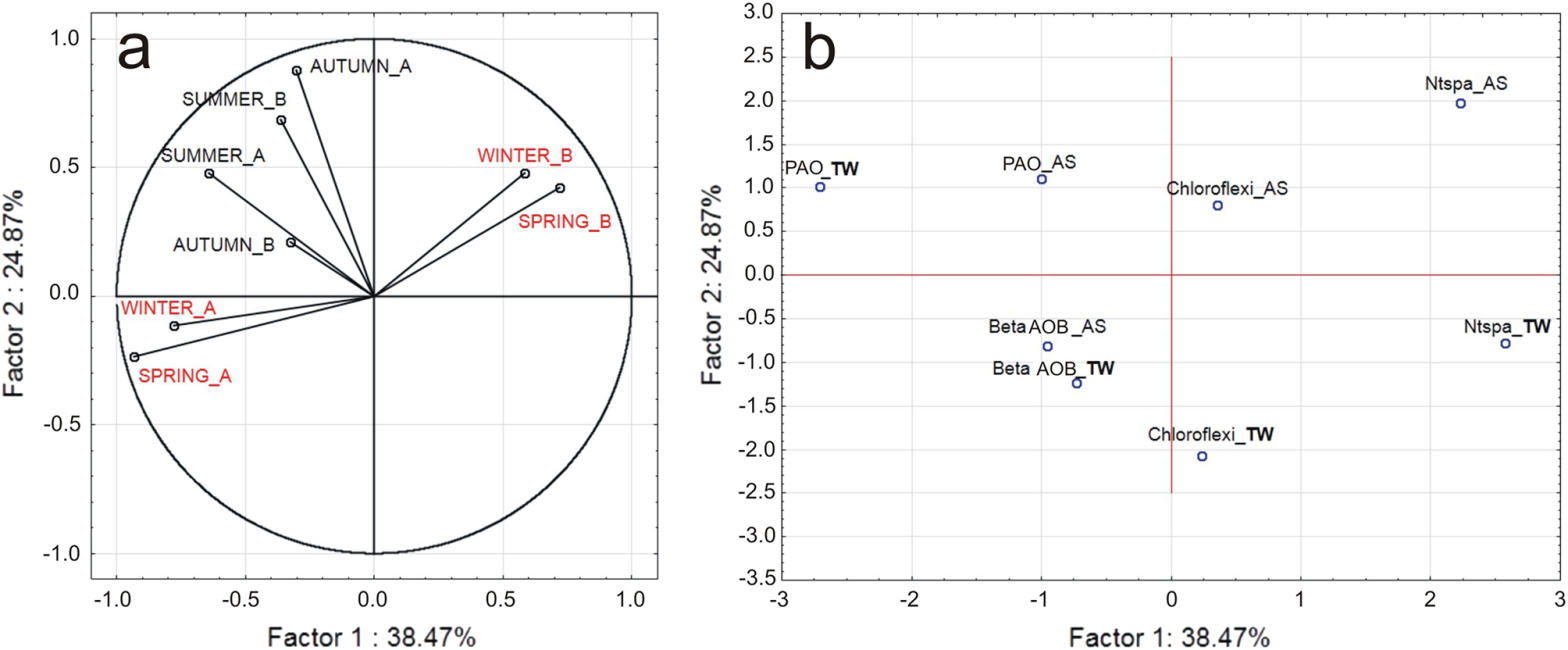
Configuration of load vectors variables (**a**) and cases (**b**) relative to the first two principal components obtained for a set of data from the quantitative analysis of bacteria from activated sludge (AS) and treated wastewater (TW) of wastewater treatment plants A and B.

Figure 4 depicts the configuration of the loading vectors with respect to the first two principal components obtained for the dataset. Data from the quantitative analysis for WWTPs A and B for different seasons indicate that the WWTPs differ (Fig. 4a). The autumn and summer periods are close to each other and occur in the first quarter. The noticeable closeness of the winter and spring periods for the same WWTPs is interesting to consider, but requires further analysis.

In most datasets, we have cases (individuals) and variables (attributes for individuals). According to configuration of load vectors (cases) (Table 4), factor 1 was under the influence of loadings obtained from abundance of Ntspa_TW and PAO_TW for A and B-WWTP. The value of factor 2 strongly correlated with loadings obtained from abundance of Chloroflexi_TW, while factor 3 was strongly influenced by loadings obtained from abundance of Beta AOB_TW. PCA analysis showed that loadings obtained from abundance of bacteria in the outflow made a larger contribution to the variance of the PC1 factorial axis than loadings obtained from abundance of bacteria from the activated sludge. Figure 4b shows that the bacteria from the outflow are located away from the center of the PC1 axis (0 value).

**Table 4:** Eigenvectors of the first three factors for the set of samples for quantitative analysis results by season (cases).

Cases	Factor 1	Factor 2	Factor 3
Beta AOB_AS	− 0.54370	− 0.57510	**1.65140**
Beta AOB_TW	− 0.41493	− 0.87908	− **1.92411**
Ntspa_AS	1.26853	**1.40046**	− 0.16310
Ntspa_TW	**1.46826**	− 0.55153	0.38124
Chloroflexi_AS	0.20266	**0.57107**	0.04470
Chloroflexi_TW	0.13386	− **1.47242**	0.13897
PAO_AS	− 0.57136	**0.78435**	− 0.49414
PAO_TW	** − 1.54332**	0.72225	0.36503

Significant values are in [bold].

During the verification, the parameters of nitrogen compounds and turbidity (N-NH_4_+, N-NO_3_^−^, N-NO_2_^−^, Turbidity) deserved special attention and were included in the further analysis. Table 5 presents the calculated eigenvalues of the 7 factors determined by the correlation matrix. For the dataset, the first factor explained 43.94% of the total variance of the dataset, and the second factor explained 20.97%. The cumulative eigenvalues of the explained variance of the first two factors was 64.92%.

**Table 5:** Eigenvalues of the correlation matrix for quantitative analysis results by season and selected physical–chemical parameters.

Factor	Eigenvalue	Cumulative eigenvalue%	Cumulative eigenvalue	Cumulative %
**1**	**3.515463**	**43.94328**	**3.515463**	**43.9433**
**2**	**1.678184**	**20.97729**	**5.193646**	**64.9206**
**3**	**1.106578**	**13.83222**	**6.300224**	**78.7528**
4	0.830157	10.37696	7.130381	89.1298
5	0.663184	8.28980	7.793565	97.4196
6	0.140738	1.75922	7.934303	99.1788
7	0.065697	0.82122	8.000000	100.0000

Factors for which cumulative eigenvalues exceeded 80 % [bold].

Table 6 shows the eigenvectors of the first tree factors with the largest contribution to the cumulative eigenvalue. Factor 1 was most influenced by loadings obtained from abundance of PAO, Chloroflexi and Nitrospirae in AS as well as PAO and Chloroflexi in TW. Factor 2 was influenced by loadings obtained from abundance of Beta AOB in the AS and Nitrospirae in the TW, as well as wastewater parameters such as NO3_AS, NH4_AS, NO2_TW, and turbidity in treated wastewater (TUR_TW). Factor 3 depended on loadings obtained from abundance of Beta AOB _TW.

**Table 6:** Eigenvectors of the first four factors for the set of samples for quantitative analysis results by season and selected physico-chemical parameters.

Cases	Factor 1	Factor 2	Factor 3
Beta AOB_AS	− 0.091549	**0.719340**	− 0.462228
Beta AOB_TW	− 0.392621	0.115007	**0.855150**
Ntspa_AS	− **0.784763**	− 0.520962	− 0.260426
Ntspa_TW	0.130682	− **0.765016**	− 0.134940
Chloroflexi_AS	− **0.874195**	− 0.339318	− 0.121694
Chloroflexi_TW	− **0.614718**	0.181738	− 0.147123
PAO_AS	− **0.902126**	0.063480	0.179857
PAO_TW	− **0.874112**	0.372371	− 0.082512
*NO3_AS	0.140299	**0.497039**	− 0.073753
*NH4_AS	0.206186	**0.583587**	0.083535
*NO2_TW	0.191554	− **0.325511**	− 0.114169
*NTU_TW	0.306957	− **0.469075**	0.239393

Significant values are in [bold].

**Figure 5:**
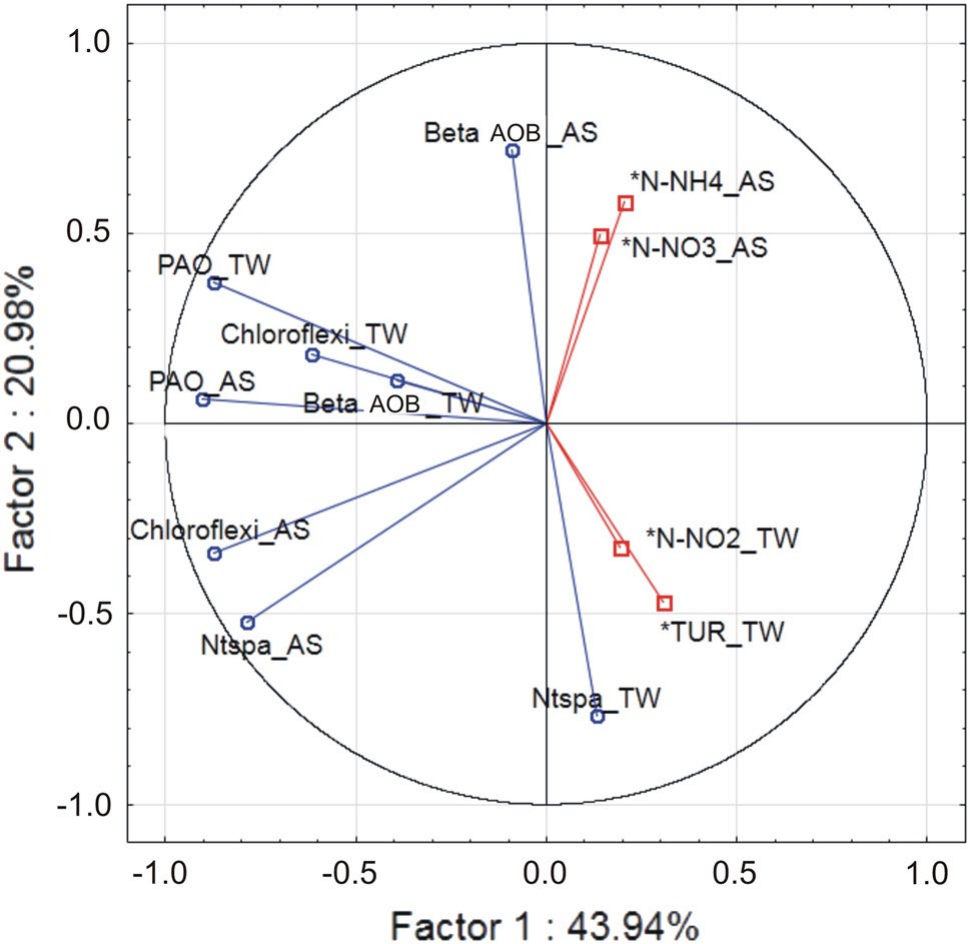
Configuration of load vectors (variables) relative to the first two principal components obtained for a set of data from the quantitative analysis of bacteria and nitrogen compounds and turbidity from activated sludge (AS) and treated wastewater (TW) of wastewater treatment plants A and B.

Figure 5 depicts the configuration of the loading vectors with respect to the first two principal components obtained for the dataset. It can be observed that ammonia oxidizing betaproteobacteria in the activated sludge (Beta AOB_AS) and Nitrospirae in treated wastewater (Ntspa_TW) have been distinguished. Beta AOB_AS are influenced by ammonia and nitrate nitrogen, while Nitrospirae in treated wastewater are dependent on nitrate nitrogen. In addition, turbidity at the outflow was related to Nitrospirae content. While the relationship of AOB bacteria (in this case, the NSO1225 probe indicates both *Nitrosomonas* and *Nitrosospira*) in the activated sludge with nitrogen compounds has been documented, data on the relationship of physico-chemical parameters at the outflow with the presence of microflora responsible for the transformation of nitrogen compounds is missing. Further research is required to find statistically significant relationships, but the results confirm the findings of the study by other authors that the unstable nitrification problem is quite common at full-scale wastewater treatment plants^56^, and that the decrease in wastewater treatment efficiency often occurs in winter^8,21^_._

Key nitrifying bacteria require extended growth periods (> 5 days) to build a functionally stable ammonia-oxidizing community, so their occurrence is often correlated with sludge retention time (SRT)^15^. In order to prevent the seasonal breakdown of nitrification, it is usual to increase the volume of biomass (mixed-liquor suspended solids (MLSS)) and extend the SRT as well as the sludge aeration period^21,57,58^. Unfortunately, these procedures are not always satisfactory. Although there are studies in the literature that confirm a direct correlation between loss of nitrifying biomass and decreasing nitrogen removal efficiency, as well as studies showing ammonia removal efficiency at low temperatures is not influenced by bacterial abundance, research by Johnston et al.^21^ suggests that the failures of nitrification at lower temperatures may be due to a different kinetic response of nitrifying taxa or other organisms with strong seasonal fluctuations in population size. On the other hand, the study of Saunders et al.^25^ indicates that not only abundant microorganisms determine the proper course of processes, but trace bacteria may also play an important role in the transformation of nitrogen and phosphorus compounds [may have a specialized but important niche, e.g. for the degradation of low concentration micropollutants]. The study of Liu et al.^59^, carried out on 8 full-scale WWTPs in China, indicated the possibility of predicting parameters such as BOD, SS, TN, TP and N-NH_4_ based on data from high-throughput sequencing. Mathematical models and bioinformatics tools^38^, e.g. using machine learning^60^, are needed to better understand the phenomenon of seasonal variation and the importance of microbial uptake in water bodies in the future. The research confirmed that data from the outflow can be a valuable source of information on the operation of wastewater treatment plants, but in order to create such models, more data needs to be collected from both activated sludge and the outflow, which has not been studied so far.

## Conclusions

The novelty of this research was to show that the sludge leaching into the outflow is a valuable source of information about the bacteria carrying out the wastewater treatment process. So far, the outflow has been studied in terms of physico-chemical parameters or pathogenic bacteria, antibiotic-resistant bacteria and also microplastics. It is advisable to continue research in the identification of bacteria associated with wastewater treatment processes in the outflow using modern molecular methods in the context of their potential technological usefulness. These additional conclusions may be drawn from the study:

1. Increased abundance of betaproteobacterial ammonia-oxidizing bacteria in the outflow in winter confirms the findings of other researchers that seasonal nitrification breakdowns occur mainly in the winter months. Comparing bacteria abundance in the activated sludge, there was no clear decrease in the abundance of these bacteria in winter for both treatment plants. Therefore, it can be noticed that the result of changes in bacteria abundance at the outflow may suggest nitrification problems, which occurred in the activated sludge chamber.
2. Nitrospirae predominated in the winter and was the lowest in summer outflow for both treatment plants.
3. During the summer, increased abundances of Chloroflexi were observed in the outflow, especially for A-WWTP; for B-WWTP were not as varied as for A-WWTP.
4. At A-WWTP, the highest abundance of *Ca.* Accumulibacter phosphatis at the outflow was observed in autumn. At the outflow of B-WWT, bacteria were scarce and there was no significant variation between samples. This may be related to the technological system, wherein biological removal of phosphorus takes place without additional support.
5. Nitrospirae, Chloroflexi and *Ca.* Accumulibacter phosphatis in treated wastewater reflect the trend in abundance of these bacteria in activated sludge.
6. Principal component analysis showed that loadings obtained from abundance of bacteria in the outflow made larger contributions to the variance in the PC1 factorial axis than loadings obtained from abundance of bacteria from the activated sludge. The results showed that the subject is worth further investigation.

## Supplementary Information

Supplementary Information.

## Data Availability

The data is included within the manuscript, and all data is fully available without restriction. Contact Magdalena Domańska for access to the data.

## References

[1] Zhang B, Yu Q, Yan G, Zhu H, Zhu L (2018). Seasonal bacterial community succession in four typical wastewater treatment plants: Correlations between core microbes and process performance. Sci. Rep..

[2] Domańska M, Wiercik P, Mituła P, Łomotowski J, Konieczny T (2020). Membrane fouling due to concentrating leachate after methane fermentation and nitrification. Desalin. Water Treat..

[3] Kuhn R, Bryant IM, Jensch R, Böllmann J (2022). Applications of environmental nanotechnologies in remediation, wastewater treatment, drinking water treatment, and agriculture. Appl. Nano.

[4] Charazińska S, Burszta-Adamiak E, Lochyński P (2021). Recent trends in Ni(II) sorption from aqueous solutions using natural materials. Rev. Environ. Sci..

[5] McIlroy SJ (2015). MiDAS: The field guide to the microbes of activated sludge. Database.

[6] Zhang T, Shao M-F, Ye L (2012). 454 Pyrosequencing reveals bacterial diversity of activated sludge from 14 sewage treatment plants. ISME J..

[7] Xu S, Yao J, Ainiwaer M, Hong Y, Zhang Y (2018). Analysis of bacterial community structure of activated sludge from wastewater treatment plants in winter. Biomed. Res. Int..

[8] Kang XH, Leng Y, O MM, Zeng XY, Li SW (2020). The seasonal changes of core bacterial community decide sewage purification in sub-plateau municipal sewage treatment plants. Bioprocess Biosyst. Eng..

[9] Nielsen PH (2010). A conceptual ecosystem model of microbial communities in enhanced biological phosphorus removal plants. Water Res..

[10] Siripong S, Rittmann BE (2007). Diversity study of nitrifying bacteria in full-scale municipal wastewater treatment plants. Water Res..

[11] Tian L, Wang L (2020). A meta-analysis of microbial community structures and associated metabolic potential of municipal wastewater treatment plants in global scope. Environ. Pollut..

[12] Seviour R, Nielsen PH (2010). Microbial ecology of activated sludge.

[13] Petrovski S, Rice DT, Batinovic S, Nittami T, Seviour RJ (2020). The community compositions of three nitrogen removal wastewater treatment plants of different configurations in Victoria, Australia, over a 12-month operational period. Appl. Microbiol. Biotechnol..

[14] Shchegolkova NM, Krasnov GS, Belova AA, Dmitriev AA, Kharitonov SL, Klimina KM, Kudryavtseva AV (2016). Microbial community structure of activated sludge in treatment plants with different wastewater compositions. Front. Microbiol..

[15] Valentin-Vargas A, Toro-Labrador G, Massol-Deya AA (2012). Bacterial community dynamics in full-scale activated sludge bioreactors: Operational and ecological factors driving community assembly and performance. PLoS One.

[16] Isazadeh S, Jauffur S, Frigon D (2016). Bacterial community assembly in activated sludge: Mapping beta diversity across environmental variables. Microbiologyopen.

[17] Gao P (2016). Correlating microbial community compositions with environmental factors in activated sludge from four full-scale municipal wastewater treatment plants in Shanghai, China. Appl. Microbiol. Biotechnol..

[18] Gonzalez-Martinez A, Rodriguez-Sanchez A, van Loosdrecht M, Gonzalez-Lopez J, Vahala R (2016). Detection of comammox bacteria in full-scale wastewater treatment bioreactors using tag-454-pyrosequencing. Environ. Sci. Pollut. Res..

[19] Gonzalez-Martinez A (2018). Microbial ecology of full-scale wastewater treatment systems in the Polar Arctic Circle: Archaea. Bacteria Fungi. Sci. Rep..

[20] Griffin JS, Wells GF (2017). Regional synchrony in full-scale activated sludge bioreactors due to deterministic microbial community assembly. ISME J..

[21] Johnston J, LaPara T, Behrens S (2019). Composition and dynamics of the activated sludge microbiome during seasonal nitrification failure. Sci. Rep..

[22] Ju F, Guo F, Ye L, Xia Y, Zhang T (2014). Metagenomic analysis on seasonal microbial variations of activated sludge from a full-scale wastewater treatment plant over 4 years. Environ. Microbiol. Rep..

[23] Ju F, Zhang T (2015). Bacterial assembly and temporal dynamics in activated sludge of a full-scale municipal wastewater treatment plant. ISME J..

[24] Muszyński A, Tabernacka A, Miłobędzka A (2015). Long-term dynamics of the microbial community in a full-scale wastewater treatment plant. Int. Biodeterior. Biodegrad..

[25] Saunders AM, Albertsen M, Vollertsen J, Nielsen PH (2016). The activated sludge ecosystem contains a core community of abundant organisms. ISME J..

[26] Amanatidou E, Samiotis G, Trikoilidou E, Tzelios D, Michailidis A (2016). Influence of wastewater treatment plants’ operational conditions on activated sludge microbiological and morphological characteristics. Environ. Technol..

[27] Janiak K, Zięba B, Szetela R, Muszyński-Huhajło M, Miodoński S, Jurga A, Trusz A (2021). Approach to modeling protozoa grazing on the basis of the current state of knowledge. Ecol. Modell..

[28] Luxmy BS, Nakajima F, Yamamoto K (2000). Predator grazing effect on bacterial size distribution and floc size variation in membrane-separation activated sludge. Water Sci. Technol..

[29] Arregui L, Linares M, Pέrez-Uz B, Guinea A, Serrano S (2008). Involvement of crawling and attached ciliates in the aggregation of particles in wastewater treatment plants. Air Soil Water Res..

[30] Paśmionka IB, Bulski K, Herbut P, Boligłowa E, Vieira FMC, Bonassa G, Bortoli M, Prá MCD (2021). Toxic effect of ammonium nitrogen on the nitrification process and acclimatisation of nitrifying bacteria to high concentrations of NH_4_-N in Wastewater. Energies.

[31] Domanska M, Kaminska J (2022). Quantification of proteobacteria with fluorescence in situ hybridization and next-generation sequencing. Environ. Eng. Manag. J..

[32] Shomar B, Al-Darwish K, Vincent A (2020). Optimization of wastewater treatment processes using molecular bacteriology. J. Water Process. Eng..

[33] Paśmionka IB, Bulski K, Herbut P, Boligłowa E, Vieira FMC, Bonassa G, Prá MCD, Bortoli M (2021). Evaluation of the effectiveness of the activated sludge process in the elimination both ATB-resistant and ATB-susceptible *E. coli* strains. Energies.

[34] De Sá LC, Oliveira M, Ribeiro F, Rocha TL, Futter MN (2018). Studies of the effects of microplastics on aquatic organisms: What do we know and where should we focus our efforts in the future?. Sci. Total Environ..

[35] Nielsen P, Daims H (2009). FISH handbook for biological wastewater treatment.

[36] Moretti G, Matteucci F, Ercole C, Vegliò F, Del Gallo M (2016). Microbial community distribution and genetic analysis in a sludge active treatment for a complex industrial wastewater: A study using microbiological and molecular analysis and principal component analysis. Ann. Microbiol..

[37] Kuśnierz M, Domańska M, Hamal K, Pera A (2022). Application of integrated fixed-film activated sludge in a conventional wastewater treatment plant. Int. J. Environ. Res. Public Health.

[38] Xia Y, Wen X, Zhang B, Yang Y (2018). Diversity and assembly patterns of activated sludge microbial communities: A review. Biotechnol. Adv..

[39] Wagner M, Loy A (2002). Bacterial community composition and function in sewage treatment systems. Curr. Opin. Biotechnol..

[40] Daims H (2015). Complete nitrification by Nitrospira bacteria. Nature.

[41] Zhang B, Xu X, Zhu L (2017). Structure and function of the microbial consortia of activated sludge in typical municipal wastewater treatment plants in winter. Sci. Rep..

[42] Roots P, Wang Y, Rosenthal AF, Griffin JS, Sabba F, Petrovich M, Wells GF (2018). Comammox Nitrospira are the dominant ammonia oxidizers in a mainstream low dissolved oxygen nitrification reactor. bioRxiv.

[43] Daims H (2006). Ecophysiology and niche differentiation of Nitrospira-like bacteria, the key nitrite oxidizers in wastewater treatment plants. Water Sci. Technol..

[44] Alawi M, Off S, Kaya M, Spieck E (2009). Temperature influences the population structure of nitrite-oxidizing bacteria in activated sludge. Environ. Microbiol. Rep..

[45] Ward LM, Hemp J, Shih PM, McGlynn SE, Fischer WW (2018). Evolution of phototrophy in the Chloroflexi phylum driven by horizontal gene transfer. Front. Microbiol..

[46] Fang D, Zhao G, Xu X, Zhang Q, Shen Q, Fang Z, Ji F (2018). Microbial community structures and functions of wastewater treatment systems in plateau and cold regions. Bioresour. Technol..

[47] Miura Y, Watanabe Y, Okabe S (2007). Significance of Chloroflexi in performance of submerged membrane bioreactors (MBR) treating municipal wastewater. Environ. Sci. Technol..

[48] Schwartz SL (2022). Novel nitrite reductase domain structure suggests a chimeric denitrification repertoire in the phylum Chloroflexi. Microbiologyopen.

[49] Łebkowska M, Załęska-Radziwił M (2016). Microorganisms. Positive and negative role in environmental engineering (Mikroorganizmy. Pozytywna i negatywna rola w inżynierii środowiska) (In Polish).

[50] Bakos V, Gyarmati B, Csizmadia P, Till S, Vachoud L, Göde PN, Wisniewski C (2022). Viscous and filamentous bulking in activated sludge: Rheological and hydrodynamic modelling based on experimental data. Water Res..

[51] Turtin I, Vatansever A, Sanin FD (2006). Phosphorus defficiency and sludge bulking. Environ. Technol..

[52] Dorofeev A, Nikolaev YA, Mardanov A, Pimenov N (2020). Role of phosphate-accumulating bacteria in biological phosphorus removal from wastewater. Appl. Biochem. Microbiol..

[53] He S, McMahon KD (2011). Microbiology of ‘Candidatus Accumulibacter’in activated sludge. Microb. Biotechnol..

[54] Muszyński A, Załęska-Radziwiłł M, Doskocz N (2018). Factors affecting Accumulibacter population structure in full-and laboratory-scale biological reactors with nutrients removal. Water Sci. Technol..

[55] Izadi P, Izadi P, Eldyasti A (2020). Design, operation and technology configurations for enhanced biological phosphorus removal (EBPR) process: a review. Rev. Environ. Sci..

[56] Tang HL, Chen H (2015). Nitrification at full-scale municipal wastewater treatment plants: Evaluation of inhibition and bioaugmentation of nitrifiers. Bioresour. Technol..

[57] Gonzalez-Martinez A (2017). Start-up and operation of an aerobic granular sludge system under low working temperature inoculated with cold-adapted activated sludge from Finland. Bioresour. Technol..

[58] Guo J (2013). The regulation and control strategies of a sequencing batch reactor for simultaneous nitrification and denitrification at different temperatures. Bioresour. Technol..

[59] Liu T, Liu S, Zheng M, Chen Q, Ni J (2016). Performance assessment of full-scale wastewater treatment plants based on seasonal variability of microbial communities via high-throughput sequencing. PLoS One.

[60] Wu J, Song C, Dubinsky EA, Stewart JR (2021). Tracking major sources of water contamination using machine learning. Front. Microbiol..

