# Supplementary material for: Seasonal prevalence of bacteria in the outflow of two full-scale municipal wastewater treatment plants
Source: Sci Rep. 2023 Jun 30;13:10608. doi: 10.1038/s41598-023-37744-3 (PMC10313732; doi:10.1038/s41598-023-37744-3)
Supplement: Supplementary file 1 — Supplementary Information. [file 41598_2023_37744_MOESM1_ESM.docx]

**Supplementary material**

**
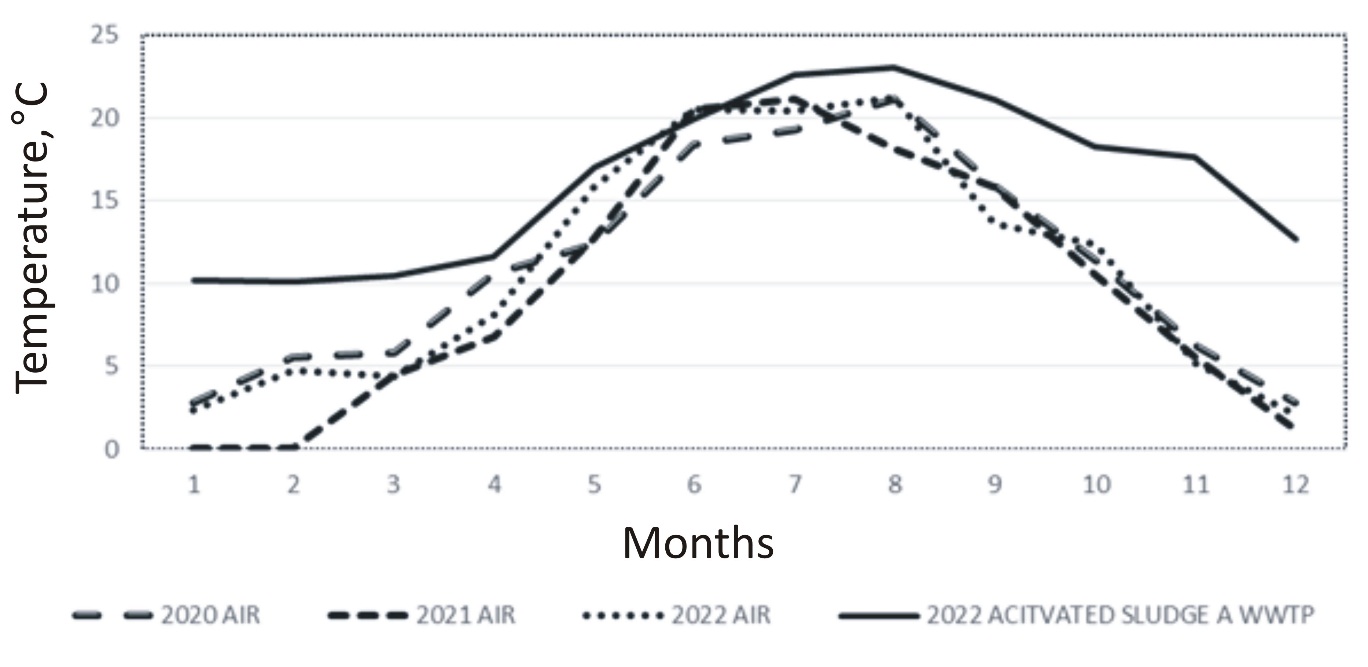
**

**Figure S1.** Average monthly air temperatures for Wroclaw in the years 2020-2022 (<https://danepubliczne.imgw.pl/>) and the instantaneous temperature measurement during wastewater sampling of the activated sludge from A-WWTP around 9 am.

**Table S1.** A list of oligonucleotide probes used in the quantitative FISH analysis [based on <https://probebase.csb.univie.ac.at/>].

| Probe | Specificity | Taxonomy | Sequence | Fluorescence dye |
| --- | --- | --- | --- | --- |
| NSO1225 | Betaproteobacterial ammonia-oxidizing bacteria | Nitrosomonadaceae; Nitrosomonadales; Betaproteobacteria; Proteobacteria; Bacteria | **5'-** CGC CAT TGT ATT ACG TGT GA **-3'** | FAM |
| Ntspa712 | most members of the phylum Nitrospirae | Nitrospirae; Bacteria | **5'-**CGC CTT CGC CAC CGG TGT TCC **-3'** | FAM |
| PAO462 | Candidatus Accumulibacter phosphatis | Candidatus Accumulibacter phosphatis; Candidatus Accumulibacter; unclassified Betaproteobacteria; Betaproteobacteria; Proteobacteria; Bacteria | **5'-** CCA TTG TAG CGT GTG TGT MG **-3'** | ROX |
| CFX1225 | phylum Chloroflexi (green nonsulfur bacteria) | Chloroflexi; Bacteria | **5'-** CCA TTG TAG CGT GTG TGT MG **-3'** | FAM |


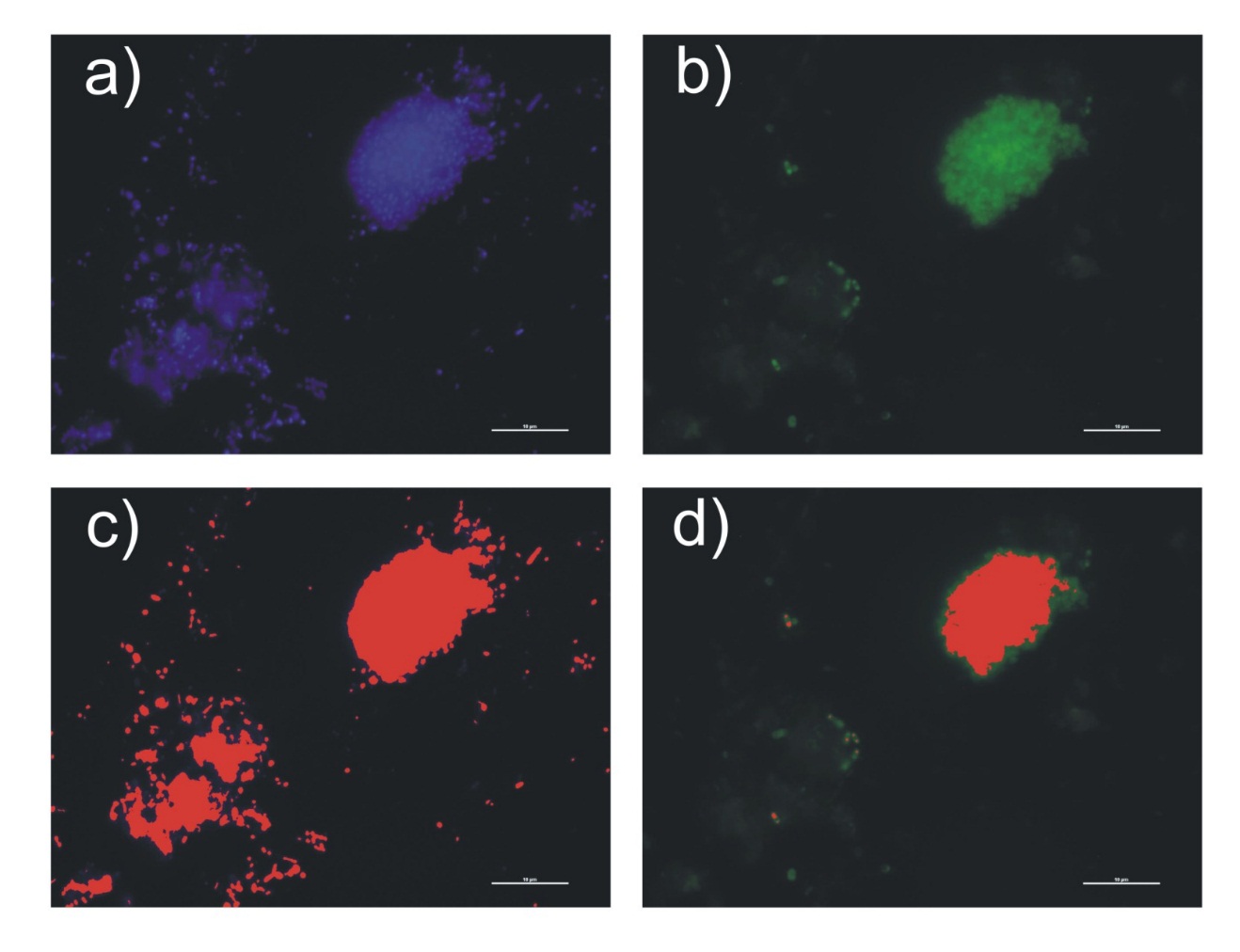


**Figure S2.** Microscopic analysis of Betaproteobacterial ammonia-oxidizing bacteria from the outflow of A-WWTP. The result of FISH staining using the NSO1225 probe (b) and DAPI (a). Photographs (c) and (d) show surfaces with the adopted threshold. Scale bar 10 μm.


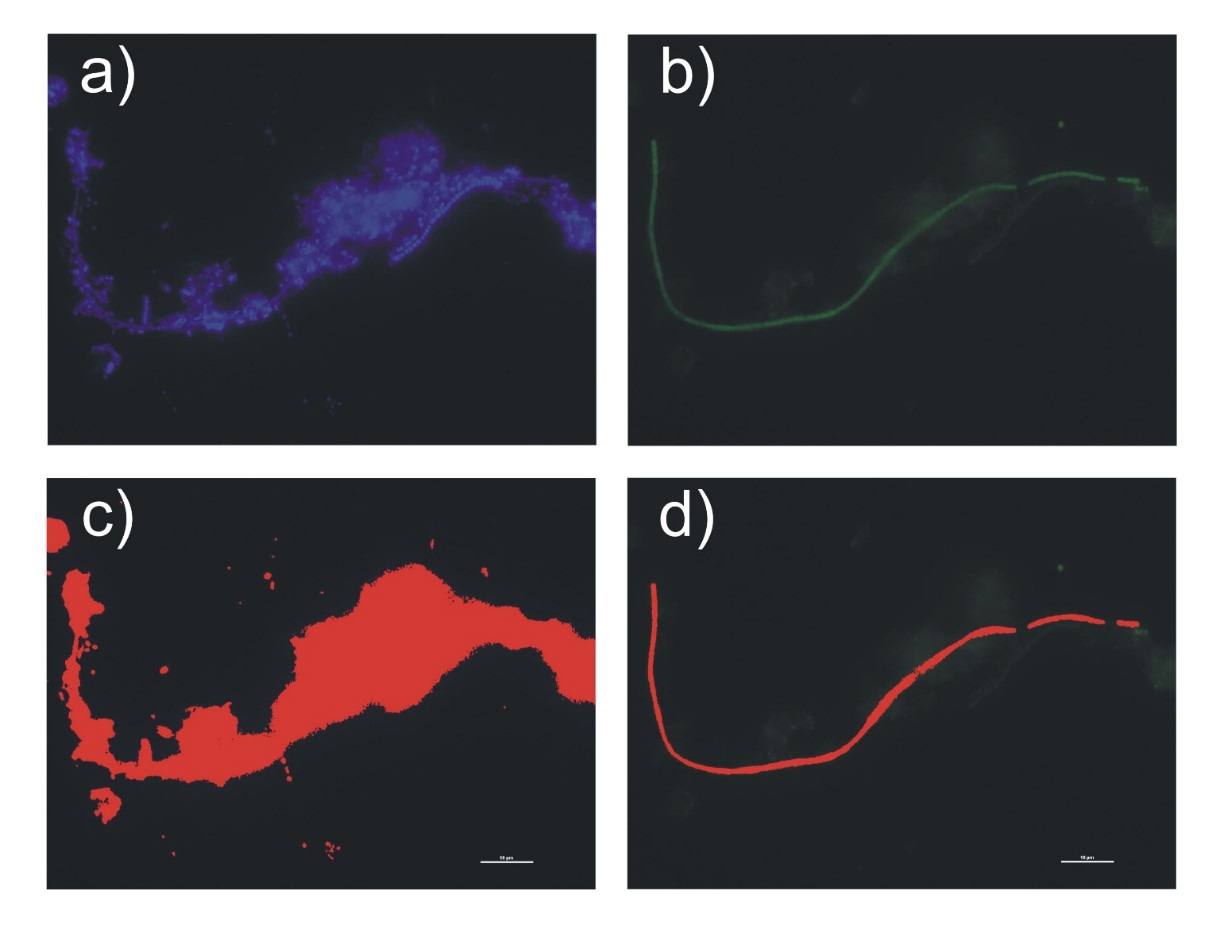


**Figure S3.** Microscopic analysis of Chloroflexi bacteria in the activated sludge from A-WWTP. The result of FISH staining using the CFX1225 probe (b) and DAPI (a). Photographs (c) and (d) show surfaces with the adopted threshold. Scale bar 10 μm.
